# The Effects of Aspirin With Combined Compound Danshen Dropping Pills on Hemorheology and Blood Lipids in Middle-Aged and Elderly Patients With CHD: A Systematic Review and Meta-Analysis

**DOI:** 10.3389/fpubh.2021.664841

**Published:** 2021-06-18

**Authors:** Zhi Liu, Gang Li, Yunwei Ma, Ling Lin

**Affiliations:** ^1^State Key Laboratory of Precision Measuring Technology and Instruments, Tianjin University, Tianjin, China; ^2^Tianjin University of Traditional Chinese Medicine, Tianjin, China; ^3^Tianjin CLINDA Medical Technology Co., Ltd., Tianjin, China

**Keywords:** compound Danshen dropping pills, aspirin, coronary heart disease, blood lipids, hemorheology, meta-analysis

## Abstract

**Background:** Coronary heart disease (CHD) is one of the most common diseases in clinical cardiovascular practice, mainly afflicting the middle-aged and elderly. It will greatly affect elderly quality of life, and even affect their psychological and physical health. At present, CHD is treated with western drugs alone, but this can produce drug dependency. In recent years, Traditional Chinese Medicine (TCM) combine western drugs has been used as a complementary and alternative therapy, and its effectiveness and safety have been proven, attracting the attention of numerous researchers.

**Objective:** Our study aimed to compare the efficacy of Aspirin with Combined Compound Danshen Dropping Pills had a superior effect on the treatment of Hemorheology and Blood Lipids in Middle-aged and Elderly Patients with CHD. Determine the effectiveness and safety of Aspirin with Combined Compound Danshen Dropping Pills in the treatment of CHD, and obtain high quality clinical evidence.

**Methods:** Based on the PRISMA Statement, inclusion and exclusion criteria were formulated. Randomized controlled trials (RCTs) on the Effects of Aspirin with Combined Compound Danshen Dropping Pills on Hemorheology and Blood Lipids in Middle-aged and Elderly Patients with CHD were found following a search of 4 mainstream medical databases. RCTs found to meet the study's requirement were included; data information was then extracted, and the quality assessed using the Cochrane bias risk assessment tool. Through RevMan software, Meta analysis was carried out for overall TC, TG, HDL-C, LDL-C hematocrit, high shear viscosity, low shear viscosity, plasma viscosity, PAGM, and TXB_2_ effective rate. The relative risk (RR) and 95% confidence interval (95% *CI*) were calculated; heterogeneity was tested and its source found; publication bias was assessed through the Egger and Begg tests, and by means of funnel plots.

**Result:** 22 RCTs were found, involving 1,987 cases. The results of the Meta analysis showed that, compared to drug therapy alone, Aspirin with Combined Compound Danshen Dropping Pills had a superior effect on the treatment of Hemorheology and Blood Lipids in Middle-aged and Elderly Patients with CHD. The meta analysis results show the effects on TC [MD = −0.91, 95% CI (−1.09, −0.73)], on TG [MD = −0.94, 95% CI (−1.22, −0.66)], on HDL-C [MD = 0.40, 95% CI (0.27, 0.53)], on LDL-C [MD = −0.99, 95% CI (−1.24, −0.74)], on hematocrit [MD = −2.69, 95% CI (−3.73, −1.65)], on high shear blood viscosity [MD = −1.11, 95% CI (−2.18, −0.05)], on low shear viscosity [MD = −0.79, 95% CI (−0.89, −0.68)], on plasma viscosity [MD = −0.26, 95% CI (−0.52, 0.01)], on PAMG [MD = −10.75, 95% CI (−16.84, −4.67)], and on TXB_2_ [MD = −11.84, 95% CI (−14.75, −8.92)]. The source of heterogeneity might be related to the state of patient, efficacy of drugs in the control group and difference in judgment criteria for efficacy. The Egger test and Begg test showed that publication bias did not occur in our study.

**Conclusions:** The combination of compound dropping pill DSP with aspirin has some therapeutic effect on blood lipids and hemorheology in patients with CHD, ince some of the RCTs featured a very small sample size, the reliability and validity of our study's conclusion may have been affected as well; therefore, the explanation should be treated with some caution. In the future, a large number of higher-quality RCTs are still needed to confirm the results of our study.

## Introduction

Coronary heart disease (CHD) is one of the most common diseases in clinical cardiovascular practice, mainly afflicting the middle-aged, and elderly. Characterized by acuteness, repeated relapse, and severe clinical manifestations, if not timely treated, it may induce myocardial infarction and sudden cardiac death. It is a life-threatening disease whose pathogenesis is related to hemadostenosis and coronary atherosclerosis (1, 2) reducing patients' quality of life (3). The key to its clinical treatment is improving myocardial blood supply, usually using antiplatelet agents. The efficacy of aspirin as an anticoagulant has been fully affirmed in clinical practice, and its price is relatively low, so it has become a first-line drug for treatment of CHD. However, it has been often reported in recent years that long-term use of antiplatelet agents can cause adverse reactions. Most patients become resistant to aspirin, weakening its efficacy (4). Therefore, it is imperative to enhance its anticoagulant effect through combination with other drugs. In elderly patients, the decline of physical function and aging progression further exacerbates various cardiovascular risk factors, making them more susceptible to CHD and other acute cardiovascular events than other age groups. Meanwhile, elderly patients are also more likely to have other systemic complications and to receive multi-drug treatment, further increasing their risk of hemorrhage during treatment with antiplatelet agents. Thus, their risk and benefits should be comprehensively evaluated prior to application to select the best therapeutic schedule to prevent the incidence of bleeding, while achieving the best curative effect (5).

According to Traditional Chinese Medicine (TCM), CHD is a symptom of “thoracic obstruction,” closely related to *qi* deficiency, blood stasis, and choroidal obstruction, so it can be treated on the principles of promoting circulation and relaxing the muscles and joints. The compound Danshen dropping pill (DSP), mainly composed of Salvia miltiorrhiza Bunge (danshen); Panax notoginseng; F.H.Chen(sanqi)and Borneol, is effective in promoting circulation, removing stasis, regulating vital energy, and easing pain, with high value for the clinical treatment of cardiovascular diseases (6). Previous studies have shown that DSP significantly improves vascular endothelial function, alleviating angina pectoris and preventing restenosis after coronary stenting, but there are relatively few reports on its influence on coronary artery lesions. Moreover, salvia miltiorrhiza, its major ingredient, can effectively control platelet activity to reduce blood viscosity, and clear out free radicals to enhance oxygen supply, so that cells and tissues can maintain normal myocardial function (7).

This paper carries out a meta-analysis on RCTs from China and elsewhere to investigate the clinical effect of DSP in combination with aspirin on CHD, aiming to provide more reliable clinical evidence for TCM.

## Materials and Methods

### Searches

Four mainstream medical databases were searched, including PubMed, CNKI, WANFANG and VIP. The timeframe used for database queries was from the earliest indexed studies to FEB 1, 2020. Search words included “Compound Danshen Dropping Pills” DSP, Danshen Dropping Pills, fufang danshen, salvia miltiorrhiza, compound red-rooted salvia, acetylsalicylic acid, Coronary Heart Disease, CHD, Blood Lipids, Hemorheology, Middle-aged, Elderly. In order to ensure the completeness of the search, the language of the database was not restricted; a search was carried out through a combination of MeSH keyword and free words; the retrieval strategy could thus be finalized (the concrete retrieval formula of the database is shown in Attachment 2). Gray literatures were manually retrieved for supplementation at the library of the Tianjin University of Traditional Chinese Medicine; if the full texts were not available, a mail was sent to its author in an attempt to obtain the full document.

### Inclusion Criteria

#### Study Types

Randomized controlled trials (RCTs) were selected, whether or not they were blinded. with no language restrictions.

#### Subjects

Patients with CHD were used, according to the CHD diagnosis criteria of the clinical diagnosis standards set by the WHO (8, 9), with average age above 50, regardless of nationality and gender, with normal hepatic and renal functions, who had not recently taken anticoagulants or antiplatelet agents.

TCM diagnosis standards: the diagnosis standards for “heart impediments” in the New Guidelines for TCM Clinical Studies (Tentative).

#### Intervention

The experimental group was treated with DSP combined with aspirin for more than 4 weeks, regardless of dosage, while the control group was treated with aspirin alone; however the use of non-anticoagulants and non-lipid-lowing drugs was allowed.

#### Primary Outcomes

Blood lipid indices: Triglyceride and total cholesterol (TC).

#### Secondary Outcomes

Blood lipid indexes: high-density lipoprotein cholesterol (HDL-C), low-density lipoprotein cholesterol (LDL-C); hemarheology indexes: hematocrit, high shear blood viscosity, low shear blood viscosity, plasma viscosity; platelet aggregation functions: thromboxane B_2_ level (TXB_2_), and maximal platelet aggregation (PAGM).

### Exclusion Criteria

The following studies were excluded from the meta-study: (1) Non-randomized trials; (2) studies lacking a control group; (3) studies reporting obviously incorrect data; and (4) duplicate studies.

### Data Selection and Data Extraction

Two researchers selected the papers independently.

First, Notexpress-2 was used for screening according to pre-set criteria, with repeated literature in the databases excluded. Then the researchers read the titles and abstracts to exclude clearly irrelevant literature.

Finally, the remaining studies were read in full, finalizing the literature selection based on the inclusion and exclusion criteria. After selection, the two researchers independently extracted the data from the included studies using Excel, including the first author's name, publication date, sample size, participants' mean age and gender, interventions in the experimental and control groups, duration of treatment, prognostic indexes, and GRADE assessment.

Adverse events were tabulated independently, and occurrences in the experimental and control groups were recorded, respectively. The adverse events collected were intestinal reactions, allergic reactions, headaches, myocardial infarctions, heart failures, cerebral hemorrhaging, cerebral infarctions, and hypertension. The two researchers cross-checked their results and discussed any issues in dispute during tabulation, resolving any outstanding issues with help from a third party.

### Quality Evaluation

The quality of the included studies was assessed in accordance with the quality assessment standards recommended in Cochrane Handbook for Systematic Reviews of Interventions 4.2.2. The assessment items included random allocation (i.e., selection bias), allocation concealment (i.e., selection bias), and blinding (i.e., performance bias). The two researchers worked independently, and resolved any issues in dispute through discussion, with outstanding issues resolved with the help of a third party.

Using the RevMan software, the bias risk assessment chart and bias risk assessment table were exported to show evaluation results for all included literature.

### Statistical Analysis

Data were extracted from the studies and entered into Excel to calculate the difference in clinical outcome between the experimental and control groups. Then, the two assessors independently entered the difference in clinical outcomes into RevMan5.3 for meta-analysis. Q test and I^2^ value were used to analyze the heterogeneity among the included studies. *P* > 0.1 and I^2^ <50% indicated a good consistency or lower heterogeneity among included studies, and a fixed effect model was adopted; otherwise, a random effect model was used. I^2^ ≤ 25% indicated a smaller heterogeneity; 25% < I^2^ ≤ 50% indicated a moderate heterogeneity, both of which could be acceptable. I^2^>50% indicated high heterogeneity between the results of the study. For higher heterogeneity, a subgroup analysis was made to explore the source of this heterogeneity; when necessary, a sensitivity analysis was made to assess the stability of the study. Through the Egger test and the Begg test carried out in the Stata software and the funnel chart, publication bias was tested. As required by the index, more than 10 pieces of literature should be included, since it would be difficult to find the reason for asymmetry if there were too few studies.

## Results

### Search Results

In the end, 22 research papers on RCTs were selected, involving 1,987 cases.

### Basic Characteristics Assessment of the Studies

**Figure 1:**
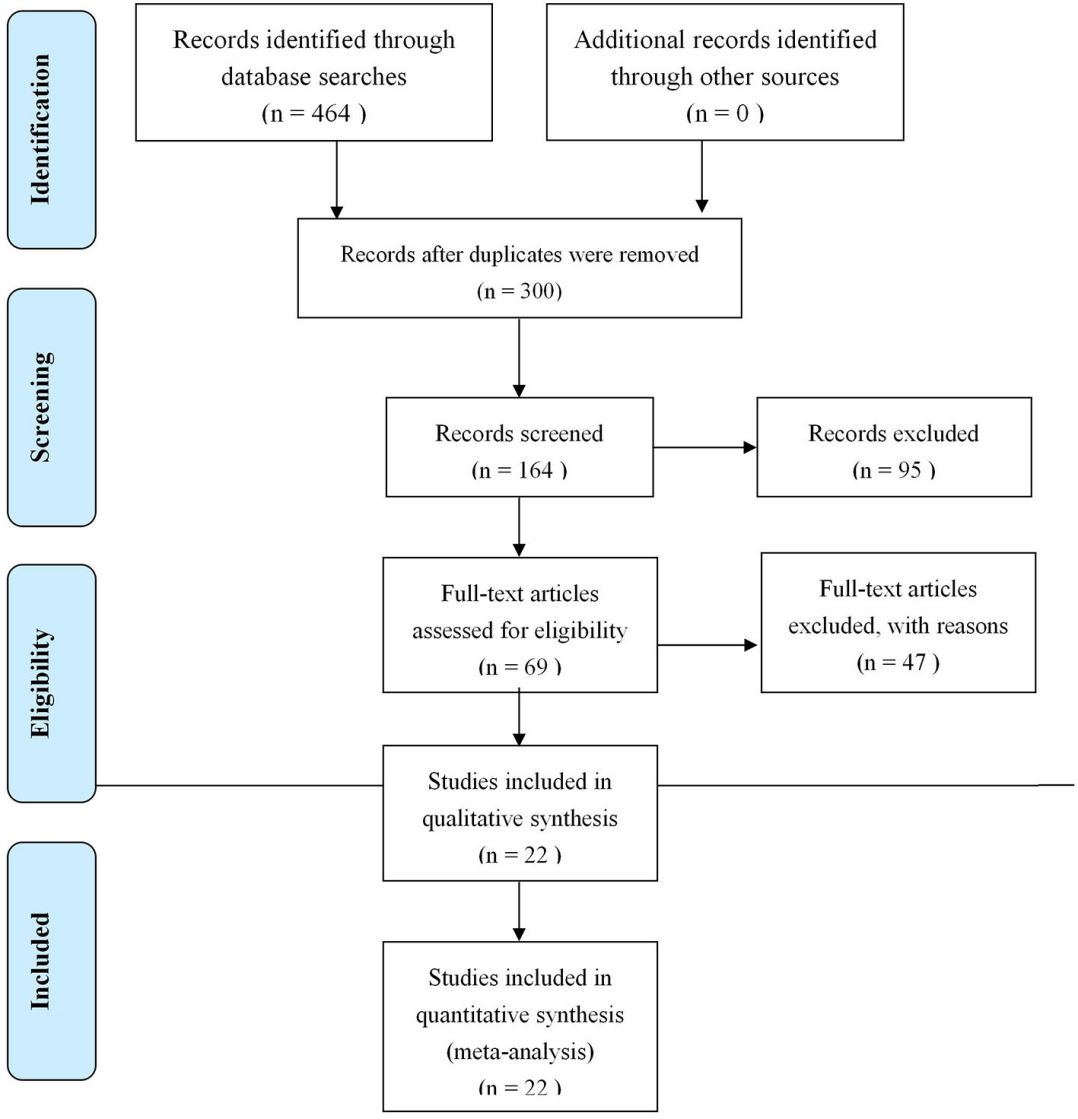
Study Screening Flow Chart.

As shown in Figure 1, 22 studies (10–31) involving 1,987 patients (1,008 experimental and 979 control) were included in the present meta-study, with sample sizes ranging from 50 to 188. All the studies were carried out in China from 2008 to 2017. The adverse events reported in five studies have no follow-up information, and all of the papers explain basic information, with no statistically significant differences regarding the patients. See Table 1 for the basic characteristics of these papers.

**Table 1:** Characteristics of the included studies.

**Study ID**	**Sample size (M/F)**		**Age (median or mean or range), y**		**Intervention**		**Course of treatment, m**	**Outcome**
	**T**	**C**	**T**	**C**	**T**	**C**		
Zhang (10)	28/17	33/12	67.4 ± 2.1	66.9 ± 2.1	Compound Danshen Dropping Pills+Asprin	Asprin	2	①②③⑤⑥⑦
Liang et al. (11)	36/23	19/11	/	/	Compound Danshen Dropping Pills+Asprin	Asprin	24	①②③④
Li (12)	29/21	28/22	/	/	Compound Danshen Dropping Pills+Asprin	Asprin	/	③④
Huang et al. (13)	30/15	27/18	59.2 ± 0.5	56.7 ± 0.3	Compound Danshen Dropping Pills+Asprin+RT	Asprin+RT	1	①②③④
Hu and Wang (14)	25/18	24/19	61.56 ± 19.25	60.25 ± 19.86	Compound Danshen Dropping Pills+Asprin	Asprin	1	①②③④
Tong (15)	14/11	14/11	/	/	Compound Danshen Dropping Pills+Asprin+RT	Asprin+RT	3	①③④
Lü et al. (16)	45/19	45/19	68.54 ± 10.58	65.98 ± 8.85	Compound Danshen Dropping Pills+Asprin	Asprin100 Mg/d	2	①②③④⑨⑩
Lin et al. (17)	20/20	22/18	50.16 ± 3.02	53.06 ± 3.10	Compound Danshen Dropping Pills+Asprin	Asprin	2	①②③④
Zhou (18)	25/21	22/24	62.4 ± 5.6	61.7 ± 4.7	Compound Danshen Dropping Pills+Asprin+RT	Asprin+RT	2	①②③④
Wang (19)	19/9	18/10	59.4 ± 6.5	59.2 ± 6.7	Compound Danshen Dropping Pills+Asprin+RT	Asprin+RT	3	①②③④
Tang et al. (20)	/	/	/	/	Compound Danshen Dropping Pills+Asprin+RT	Asprin+RT	3	①②③④
Wang (21)	18/13	17/14	54.7 ± 1.2	56.9 ± 1.5	Compound Danshen Dropping Pills+Asprin	Asprin+RT	/	①②③④⑨⑩
Wang (22)	/	/	/	/	Compound Danshen Dropping Pills+Asprin+RT	Asprin	2	①②③④
Yang (23)	20/10	18/12	55.8 ± 8.9	54.6 ± 8.2	Compound Danshen Dropping Pills+Asprin+RT	Asprin+RT	1	①②③④
Xu (24)	/	/	/	/	Compound Danshen Dropping Pills+Asprin	Asprin	2	①②③④⑤⑥⑦⑧
Xiong (25)	/	/	/	/	Compound Danshen Dropping Pills+Asprin	Asprin	6	①②③④⑤⑥⑦
Sun (26)	21/18	19/20	/	/	Compound Danshen Dropping Pills+Asprin	Asprin	2	③④
Chen (27)	34/26	35/25	/	/	Compound Danshen Dropping Pills+Asprin+RT	Asprin+RT	3	①②③④
Lin and Zhao (28)	/	/	/	/	Compound Danshen Dropping Pills+Asprin	Asprin	2	③④⑧⑨
Hong (29)	28/36	28/36	58.9 ± 3.1	59.1 ± 3.0	Compound Danshen Dropping Pills+Asprin	Asprin	2	③④
Guo et al. (30)	25/5	23/7	61.4 ± 10.5	56.8 ± 13.4	Compound Danshen Dropping Pills+Asprin+RT	Asprin+RT	1	⑨⑩
Liang et al. (31)	39/21	33/27	51.29 ± 3.38	50.19 ± 4.02	Compound Danshen Dropping Pills+Asprin+RT	Asprin+RT	6	⑨⑩

*TG(Triglycerides) TC(Total serum cholesterol) HDL-C(High-density lipoprotein cholesterol) LDL-C(Low-density lipoprotein cholesterol) Hematocrit Whole Blood Viscosity at High Shear Low Shear Blood Viscosity Plasma Viscosity PAGM(Maximum platelet aggregation rate) TXB_2_(Thromboxane B2)*.

### Summary of Test Quality and Bias Risks

**Figure 2:**
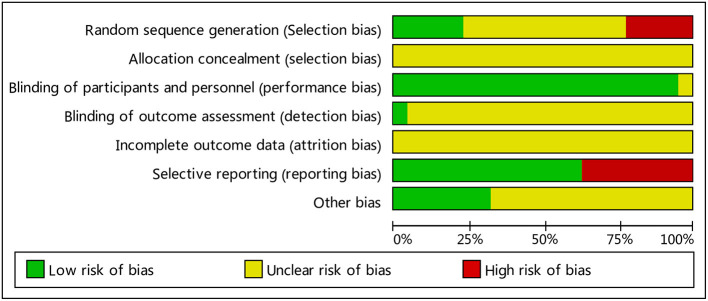
Bias risk map: the reviewers' judgment of each type of bias risk, expressed as a percentage of all included studies.

**Figure 3:**
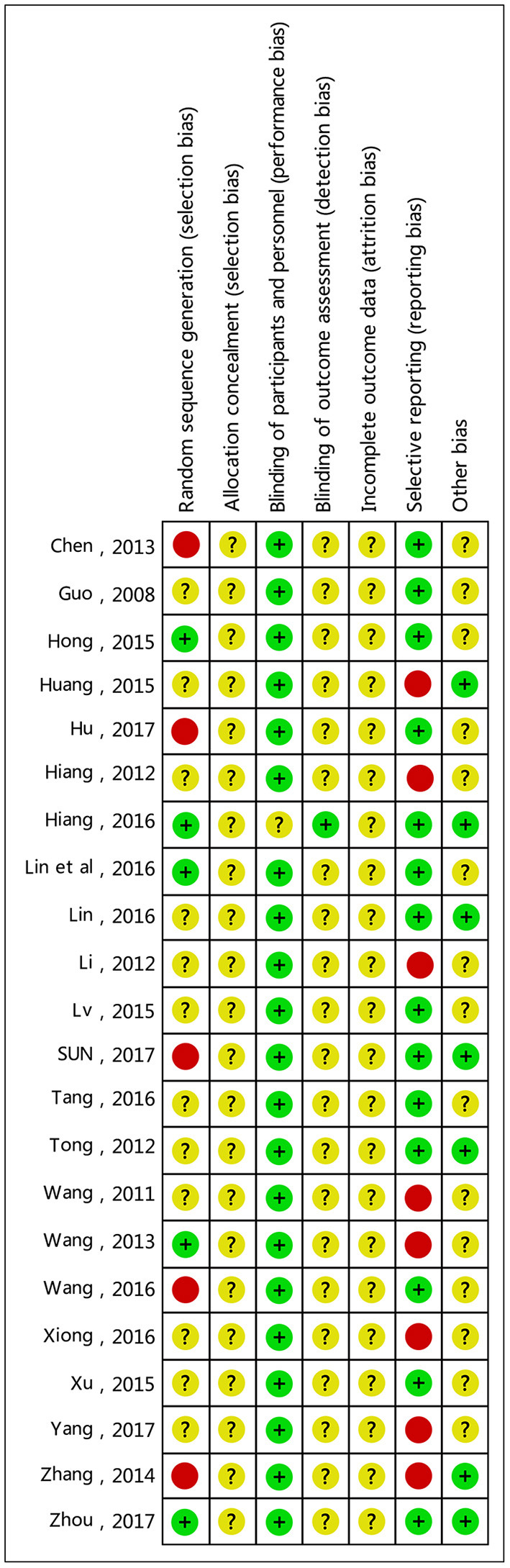
Summary of bias risks: the reviewers' judgment of risk for each bias item in each included study.

Two independent researchers evaluated the methodology, quality and all the methods in the included studies in accordance with the Cochrane handbook; any disputed issue was solved through discussion. Twenty two of the papers were low-quality. All papers had separate groups; ten reported the specific grouping method (correct grouping methods were used in five papers, while an improper method was used in another five papers); the other 12 only analyzed grouping, but did not describe the grouping method in detail. They included no information about loss to follow-up or withdrawal. All studies failed to note the blinding of participants and researchers. See Figures 2, 3 for summaries of the quality and bias risks of the included studies.

### Total Cholesterol (TC)

**Figure 4:**
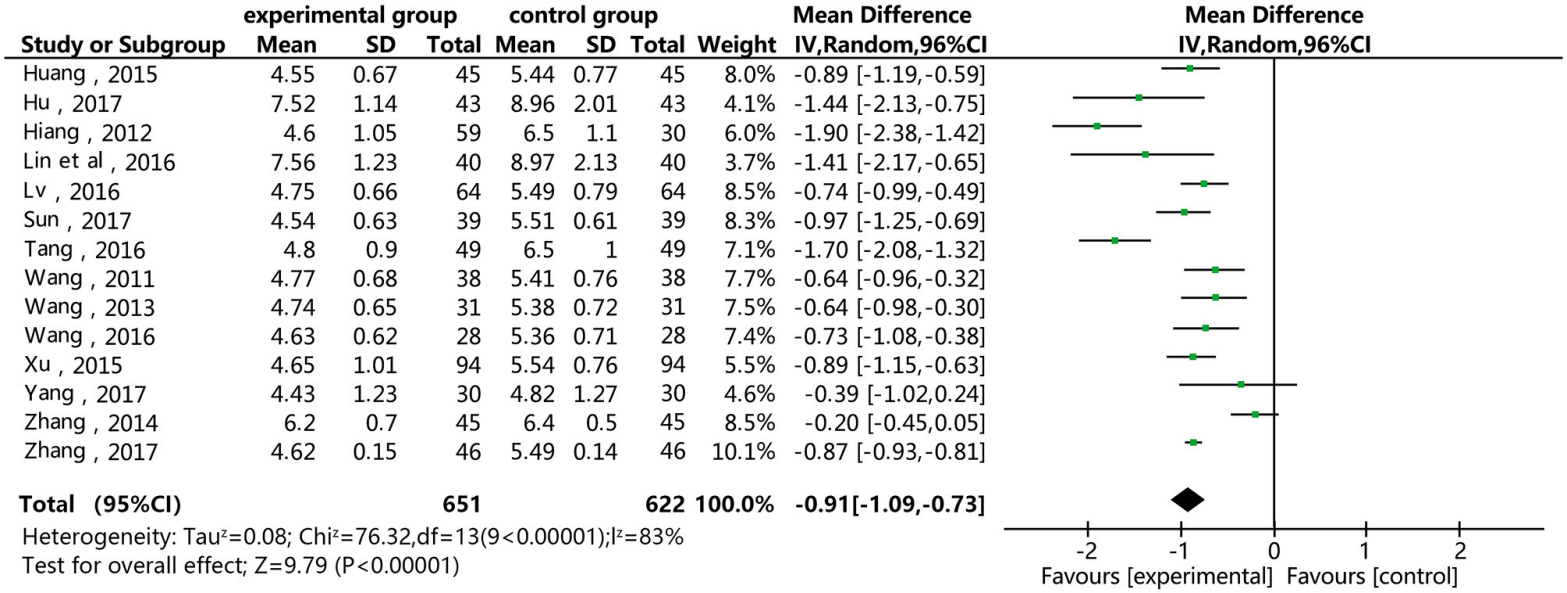
Meta-analysis forest map for the of the effect of DSP combined with aspirin on TC in patients with CHD.

Figure 4 shows that 14 of the included studies report the effect of DSP combined with aspirin on TC in patients with CHD. These studies included 1,273 cases – 651 in the experimental group, and 622 in the control. The heterogeneity test showed statistical significance (*P* < 0.00001, I^2^ = 83%), so the random effect model was used for meta-analysis. The results showed high heterogeneity between the studies, so we conducted a sensitivity analysis (see Annex 1), finding relatively high heterogeneity in one paper (18). On the premise of not removing this paper, we conducted subgroup analysis (see Annex 2), still finding high heterogeneity. We determined that this might be related to the quality of the included papers. The results showed statistical differences between the two groups [MD = −0.91, 95% CI (−1.09, −0.73)] (see Figure 4). The statistical results show that the combination of DSP with aspirin was effective in reducing TC in the patients with CHD. The quality of this part was very low according to the GRADE standard (see Table 2).

**Table 2:** GRADE quality of evidence summary table for the comparisons of Fufangdanshen Dripping combined with Aspirin vs. Aspirin for CHD.

**Outcomes**	Illustrative comparative risks^*^ **(95% CI)**		**Relative effect (95% CI)**	**No of Participants (studies)**	**Quality of the!!break evidence!!break (GRADE)**	**Comments**
	**Assumed risk**	**Corresponding risk**				
	**Asprin**	**Asprin comparisons Compound Danshen Dropping Pills**				
TC	The mean TC in the intervention groups was 0.91 lower (1.09–0.73 lower)			1,273 (14 studies)	⊕⊖⊖⊖ **very low**^1, 2, 3, 4^	
TG	The mean tg in the intervention groups was 0.94 lower (1.22–0.66 lower)			1,323 (15 studies)	⊕⊖⊖⊖ **very low**^1, 4, 5^	
HDL-C	The mean hdl-c in the intervention groups was 0.4 higher (0.27–0.53 higher)			1,679 (19 studies)	⊕⊖⊖⊖ **very low**^1, 4^	
LDL-C	The mean ldl-c in the intervention groups was 0.99 lower (1.24–0.74 lower)			1,589 (18 studies)	⊕⊖⊖⊖ **very low**^1, 4^	
Hematocrit	The mean Hematocrit in the intervention groups was 2.71 lower (3.77–1.66 lower)			337 (3 studies)	⊕⊕⊕⊖ **moderate**^5^	
Whole Blood Viscosity at High Shear	The mean Whole Blood Viscosity at High Shear in the intervention groups was 1.11 lower (2.18–0.05 lower)			465 (4 studies)	⊕⊖⊖⊖ **very low**^1, 4, 5^	
Low Shear Blood Viscosity	The mean Low Shear Blood Viscosity in the intervention groups was 0.79 lower (0.89–0.68 lower)			465 (4 studies)	⊕⊖⊖⊖ **very low**^1, 2, 3^	
Plasma Viscosity	The mean Plasma Viscosity in the intervention groups was 0.26 lower (0.52 lower to 0.01 higher)			308 (3 studies)	⊕⊖⊖⊖ **very low**^1, 2^	
PAGM	The mean PAGM in the intervention groups was 7.69 lower (8.87–6.5 lower)			406 (4 studies)	⊕⊖⊖⊖ **very low**^1, 2, 3^	
TXB_2_	The mean TBX_2_ in the intervention groups was 11.84 lower (14.75–8.92 lower)			406 (4 studies)	⊕⊕⊕⊖ **moderate**^2^	

* *the basis for the assumed risk (e.g., the median control group risk across studies) is provided in footnotes. The corresponding risk (and its 95% confidence interval) is based on the assumed risk in the comparison group and the relative effect of the intervention (and its 95% CI)*.

*CI, Confidence interval*.

*GRADE Working Group grades of evidence*.

*High quality: Further research is very unlikely to change our confidence in the estimate of effect*.

*Moderate quality: Further research is likely to have an important impact on our confidence in the estimate of effect and may change the estimate*.

*Low quality: Further research is very likely to have an important impact on our confidence in the estimate of effect and is likely to change the estimate*.

*Very low quality: We are very uncertain about the estimate*.

*1. High heterogeneity value*.

*2. Lack of blinding*.

*3. Lack of allocation hiding*.

*4. Lack of allocation hiding and blinding*.

*5. Wide confidence interval leads to a decline in the quality of the evidence*.

*⊖TG(Triglycerides) 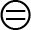
TC(Total serum cholesterol) ⊛HDL-C(High-density lipoprotein cholesterol) ④LDL-C(Low-density lipoprotein cholesterol) ⑤Hematocrit ⑥Whole Blood Viscosity at High Shear ⑦Low Shear Blood Viscosity ⑧Plasma Viscosity ⑨PAGM ⑩TXB_2_*.

### Triglycerosis (TG)

**Figure 5:**
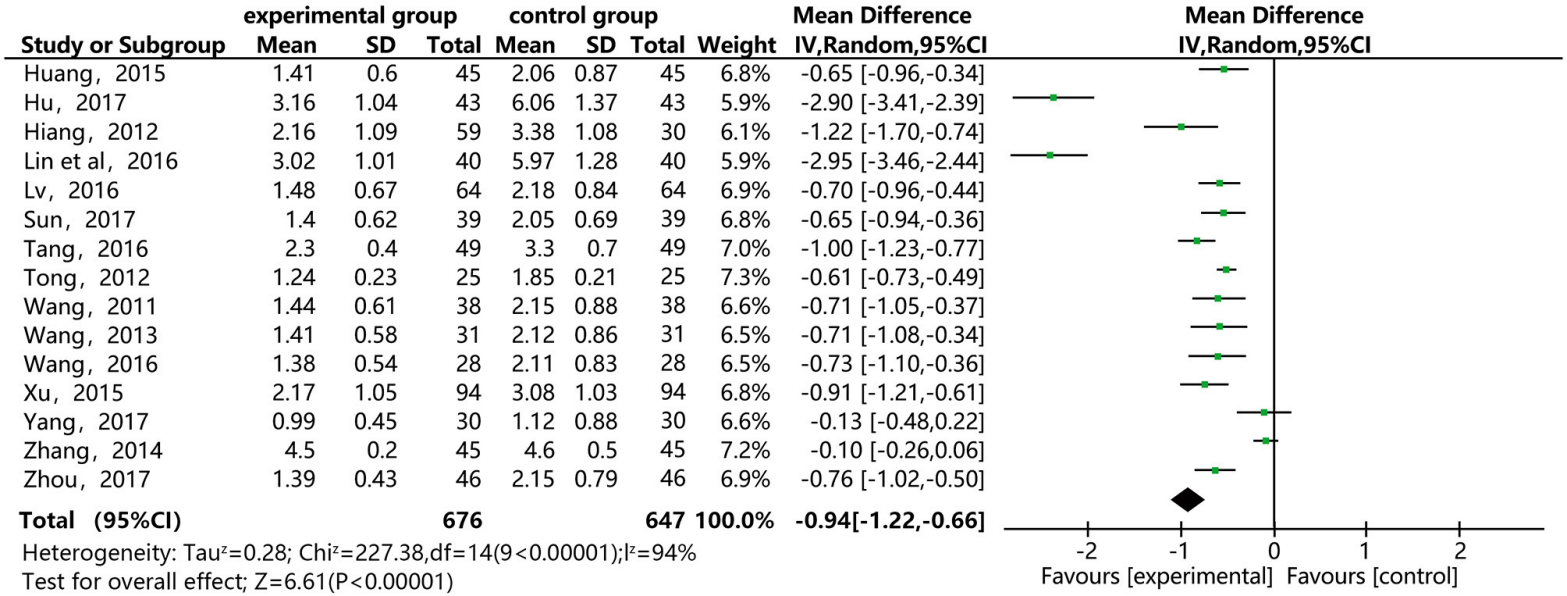
Meta-analysis forest map for the effect of DSP combined with aspirin on TG in patients with CHD.

Figure 5 shows that 15 of the included papers reported the effect of DSP combined with aspirin on TG in patients with CHD. These papers included 1,323 cases: 676 in the experimental group, and 647 in the control. The heterogeneity test showed statistical significance (*P* < 0.00001, I^2^ = 94), so the random effect model was used. The meta-analysis found high heterogeneity between the studies. A sensitivity analysis (see Annex 3) found relatively high heterogeneity in two papers (14, 17). On the premise of not removing these papers, we conducted subgroup analysis (see Annex 4), still finding high heterogeneity, which we determined may be related to the quality of the papers. The results showed statistical differences between the two groups [MD = −0.94, 95% CI (−1.22, −0.66)] (see Figure 5). The statistical results show that the combination of DSP with aspirin was effective in reducing TG in patients with CHD. The quality of this part was very low according to the GRADE standard (see Table 2).

### HDL-C

**Figure 6:**
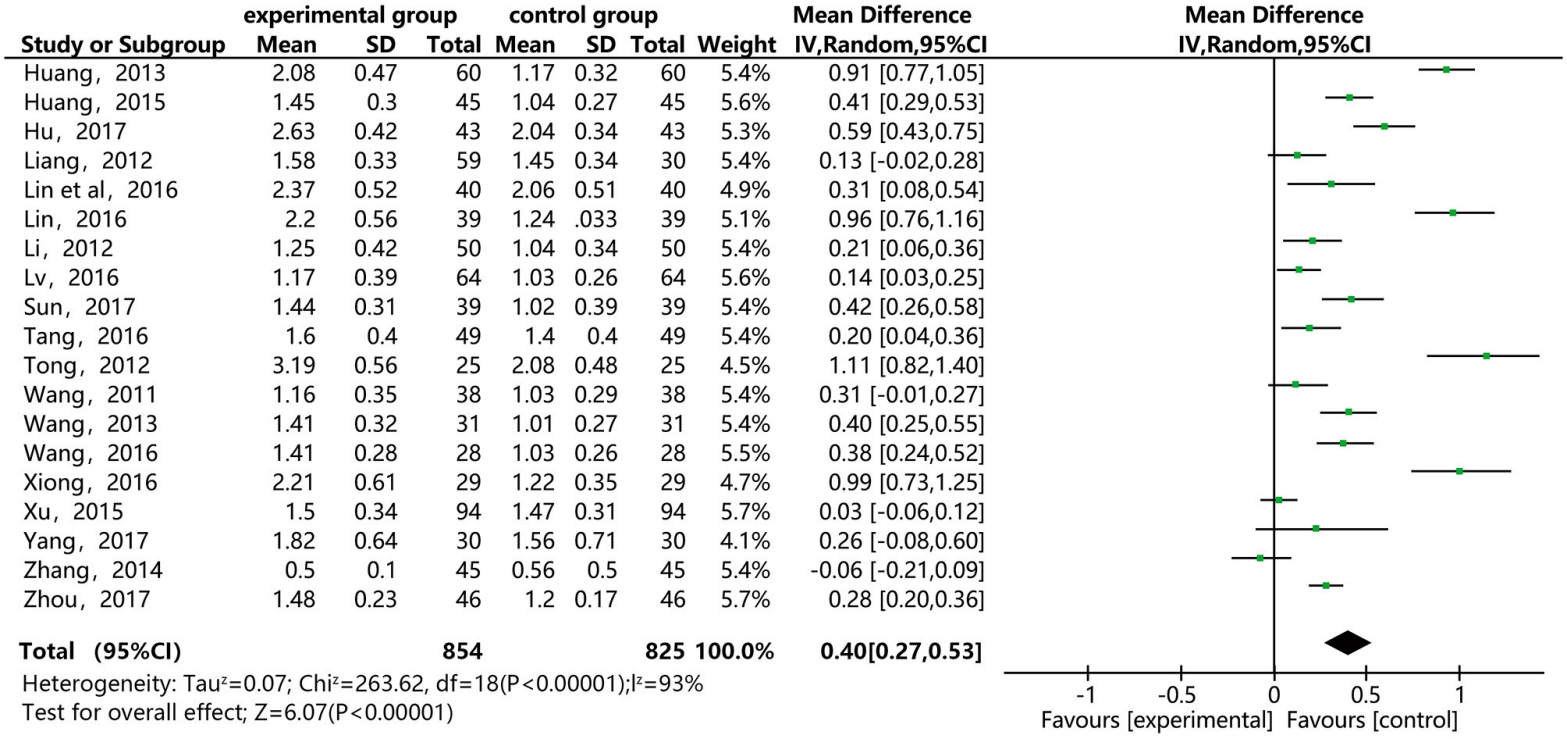
Meta-analysis forest map for of the effect of DSP combined with aspirin on HDL-C in patients with CHD.

Figure 6 shows that 19 of the included papers reported on the effect of DSP combined with aspirin on HDL-C in patients with CHD. Thousand six hundred and seventy nine cases were included: 854 in the experimental group, and 825 in the control. The heterogeneity test between the studies was statistically significant (*P* < 0.00001, I^2^ = 93%), so the random effect model was used. The analysis results showed high heterogeneity between the studies. After a sensitivity analysis (see Annex 5) and subgroup analysis (see Annex 6), there was still relatively high heterogeneity, which we ascribed to the quality of the included papers. The results showed statistically significant differences between the groups [MD = 0.40, 95% CI (0.27, 0.53)] (see Figure 6). The results showed that that the combination of DSP with aspirin was not effective in raising HDL-C in patients with CHD. The quality of this part was very low according to the GRADE standard (see Table 2).

### LDL-C

**Figure 7:**
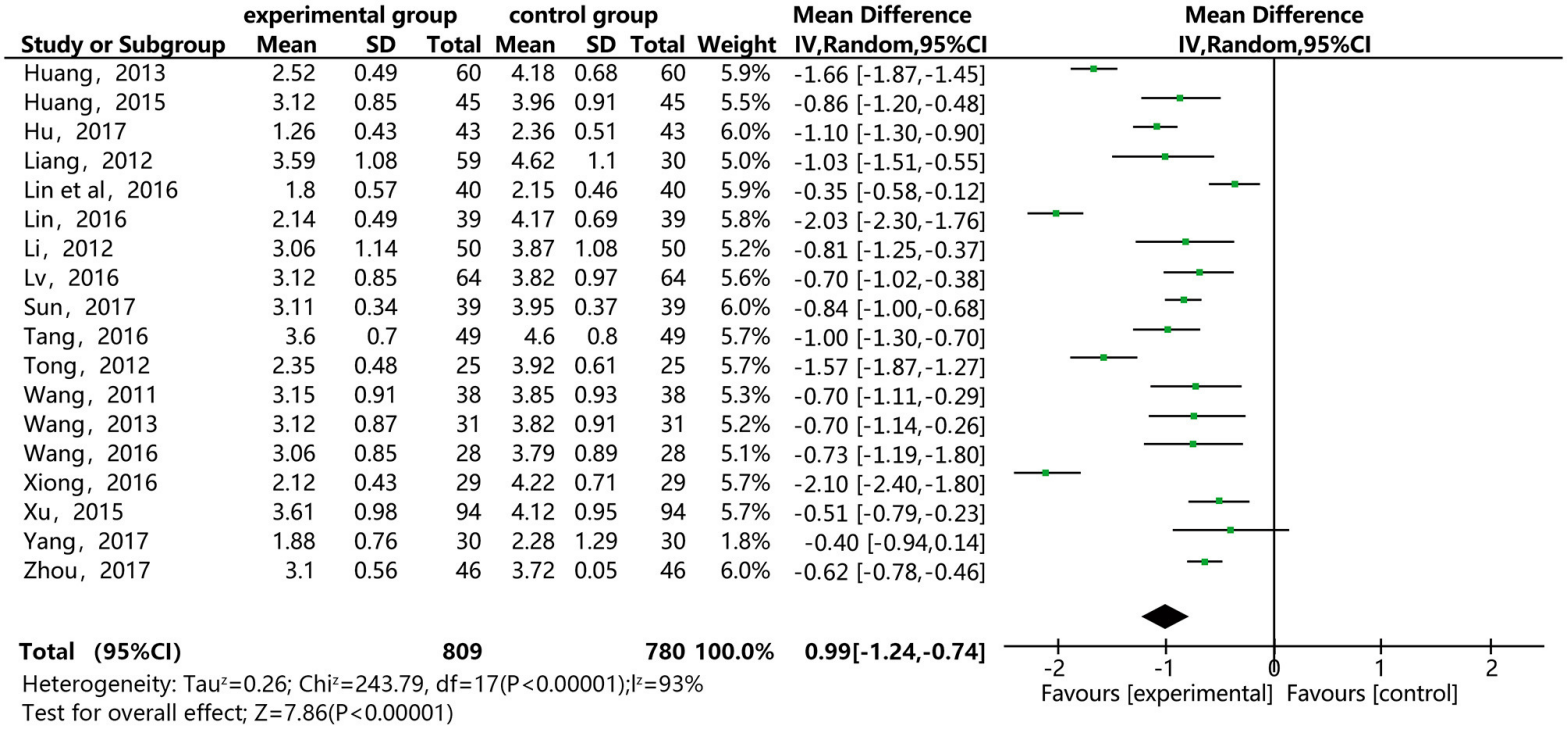
Meta-analysis forest map for the effect of DSP combined with aspirin on LDL-C in patients with CHD.

Figure 7 shows that 18 of the papers reported the effect of DSP combined with aspirin on LDL-C in patients with CHD. The studies included 1,589, with 809 in the experimental group and 780 in the control. The heterogeneity test showed statistical significance (*P* < 0.00001, I^2^ = 93%), so the random effect model was used for meta-analysis. The results found high heterogeneity between the studies. After a sensitivity analysis (see Annex 7) and subgroup analysis (Annex 8), there was still relatively high heterogeneity, which we ascribed to the quality of the papers. The results showed statistical differences between the groups [MD = −0.99, 95% CI (−1.24, −0.74)] (see Figure 7). The results of the meta-analysis show that that the combination of DSP with aspirin was effective in reducing LDL-C in patients with CHD. The quality of this part was very low according to the GRADE standard (see Table 2).

### Hematocrit

**Figure 8:**
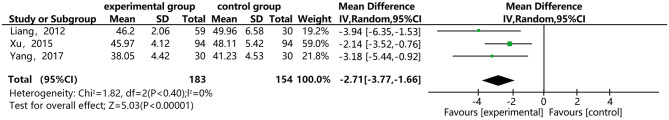
Meta-analysis forest map for the effect of DSP combined with aspirin on hematocrit in patients with CHD.

As shown in Figure 8, three of the papers reported the effect of DSP combined with aspirin on hematocrit in patients with CHD. The studies included 337 cases: 183 in the experimental group and 154 in the control. There was no heterogeneity between the studies (*P* = 0.40, I^2^ = 0%), thus, the random effect model was used for meta-analysis. The results showed statistical differences between the groups [MD = −2.69, 95% CI (−3.73, −1.65)] (see Figure 8). The results of the meta-analysis showed that that the combination of DSP with aspirin was effective in reducing hematocrit in patients with CHD. The quality of this part was very low according to the GRADE standard (see Table 2).

### High Shear Blood Viscosity

**Figure 9:**
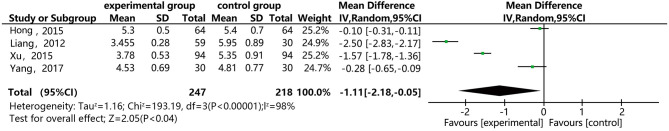
Meta-analysis forest map of the effect of DSP combined with aspirin on high shear blood viscosity in patients with CHD.

Figure 9 shows that four of the papers report the effect of DSP combined with aspirin on high shear blood viscosity at in patients with CHD. The studies include 465 cases: 247 in the experimental group and 218 in the control. There was heterogeneity between the studies (*P* < 0.00001, I^2^ = 98%), so the random effect model was used for meta-analysis. After a subgroup analysis (see Annex 9), there was still relatively high heterogeneity, which we ascribed to the quality of the included papers. The results showed statistical differences between the groups [MD = −1.11, 95% CI (−2.18, −0.05)] (see Figure 9). The meta-analysis results showed that that the combination of DSP with aspirin was effective in reducing high shear blood viscosity in patients with CHD. The quality of this part was very low according to the GRADE standard (see Table 2).

### Low Shear Blood Viscosity

**Figure 10:**
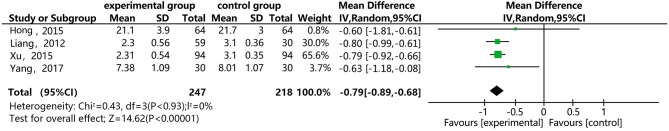
Meta-analysis forest map for the effect of DSP combined with aspirin on low shear blood viscosity in patients with CHD.

Figure 10 shows that four of the papers reported the effect of DSP combined with aspirin on low shear blood viscosity in the patients with CHD. The studies included 465 cases: 247 in the experimental group and 218 in the control group. There was no heterogeneity between the studies (*P* = 0.93, I^2^ = 0%), thus a random effect model was used for meta-analysis. The result showed statistical differences between the groups [MD = −2.69, 95% CI (−3.73, −1.65)] (see Figure 10). The meta-analysis results showed that that the combination of DSP with aspirin was effective in reducing low shear blood viscosity in the patients with CHD. The quality of this part was very low according to the GRADE standard (see Table 2).

### Plasma Viscosity

**Figure 11:**
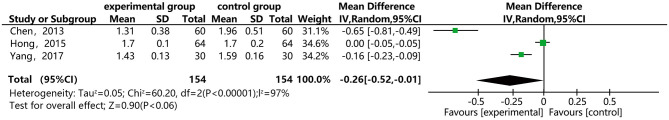
Meta-analysis forest map for the effect of DSP combined with aspirin on plasma viscosity in patients.

Figure 11 shows that three of the papers reported the effect of DSP combined with aspirin on plasma viscosity in patients with CHD. They included 308 cases: 154 in the experimental group and 154 in the control. The heterogeneity test between the studies showed statistical significance (*P* < 0.00001, I^2^ = 97%), so the random effect model was used for meta-analysis. The result showed statistical differences between the groups [MD = −0.26, 95% CI (−0.52, 0.01)] (see Figure 11). The meta-analysis showed that that the combination of DSP with aspirin was effective in reducing plasma viscosity in patients with CHD. The quality of this part was very low according to the GRADE standard (see Table 2).

### Maximal Platelet Aggregation (PAMG)

**Figure 12:**
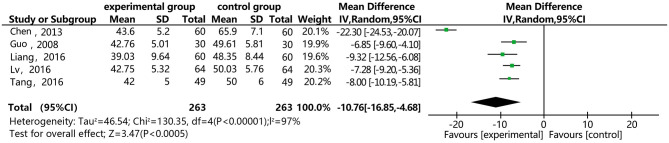
Meta-analysis forest map of the effect of DSP combined with aspirin on PAMG in patients with CHD.

Figure 12 shows that five of the papers reported the effect of DSP combined with aspirin on PAMG in the CHD patients. They included 526 cases: 263 in the experimental group and 263 in the control. The heterogeneity test was statistically significant (*P* < 0.00001, I^2^ = 97%), so the random effect model was used for meta-analysis. We conducted a sensitivity analysis, finding relatively high heterogeneity in one paper (27), and then conducted subgroup analysis (see Annex 10). We removed this paper, finding that heterogeneity I^2^ = 0%, which might be related to its quality. The results showed statistical differences between the groups [MD = −10.75, 95% CI (−16.84, −4.67)] (see Figure 12). The meta-analysis showed that that the combination of DSP with aspirin was effective in reducing PAMG in patients with CHD. The quality of this part was very low according to the GRADE standard (see Table 2).

### Thromboxane B_2_ (TXB_2_)

**Figure 13:**
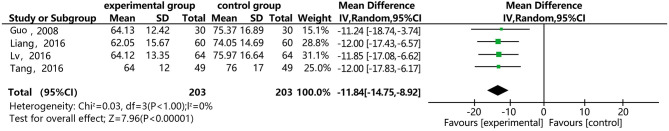
Meta-analysis forest map for the effect of DSP combined with aspirin on TXB_2_ in patients with CHD.

Figure 13 shows that 4 of the papers reported the effect of DSP combined with aspirin on TXB_2_ in patients with CHD. The studies included 406 cases: 203 in the experimental group, and 203 controls. The heterogeneity test showed statistical significance (*P* = 1.00, I^2^ = 0%), thus the random effect model was used for meta-analysis. The results showed statistical differences between the groups [MD = −11.84, 95% CI (−14.75, −8.92)] (see Figure 13). The meta-analysis showed that that the combination of DSP with aspirin was effective in reducing TXB_2_ in patients with CHD. The quality of this part was very low according to the GRADE standard (see Table 2).

## Discussion

CHD is frequently encountered in clinical practice, primarily in middle-aged and elderly patients, with angina pectoris as a common clinical symptom (32). It also has great impact on cardiovascular and other tissues. Its aggravation can induce significant complications, such as myocardial infarction and thrombosis, posing a serious threat to life. Thus, great attention should be paid to CHD. Conservative therapy is usually used to treat CHD in current clinical practice, using Western antiplatelet agents as major therapeutic drugs. Aspirin is an effective antithrombotic drug used to treat the secondary complications of primary atherosclerotic and atherosclerosis thrombotic diseases (32, 33). Studies have shown that regular use of aspirin can effectively reduce the occurrence of myocardial infarction, ischemic stroke, and lethal coronary artery diseases (34), but in recent years there have been reports of resistance to antiplatelet agents, including aspirin, and increased dosage causing severe gastrointestinal reactions. Thus, its efficacy has been questioned. The main ingredients of DSP include salvia miltiorrhiza, pseudo-ginseng, and borneol (35). Salvia miltiorrhiza is effective in promoting blood circulation and dispersing stasis, and has been proved by modern pharmacological research to inhibit platelet adhesion and aggregation, reduce blood viscosity, accelerate red blood cell flow, regulate internal and external blood coagulation, and promote fiber dissolution. Pseudo-ginseng is effective in tonifying *qi* and blood, easing pain, eliminating blood stasis, reducing blood sugar and lipids, and improving immunity. Borneol is effective against inflammation and bacteria, improving coronary flow and reducing myocardial consumption of oxygen (36).

At present, clinical trial methods for various clinical treatments of CHD (including: TCM treatment) are not of high quality, and clinical evidence is weak. On this basis, a Meta analysis was carried out in our study. Studies have shown that the combination of DSP with aspirin can effectively treat CHD and reduce adverse reactions in patients, improving to their prognosis (37). In this study, in patients treated with DSP combined with aspirin, HDL-C rose to a normal level, and LDL-C decreased to a normal level, achieving a better effect than treatment with aspirin alone. Previous research has shown that DSP, which has high stability and is not easily decomposed, can act for a long time to effectively regulate blood lipids in patients. In this study, TC, TG and LDL-C decreased more significantly in the patients treated with DSP combined with aspirin, than in those treated by aspirin alone (38). Where difference was statistically significant. However, since the included studies were of lower quality and higher heterogeneity, these results should be treated with a certain amount of caution. In recent years, numerous studies on combine therapy in Middle-aged and Elderly Patients with CHD have been published. For example: in a study of Meta analysis of Meta Analysis Based on Efficacy and Safety of Aspirin and Clopidogrel in the Treatment of Elderly Patients with Coronary Heart Disease, this combined therapy was effective and safe for CHD (39). Compared with this study, our study possessed two features: (1)Because clopidogrel and aspirin are prone to dependence after long-term service, Chinese patent medicine combined with western medicine has higher clinical safety than two kinds of western medicine, which is more suitable for clinical promotion. (2) Our study involved more literature review (18 vs. 22). However, publication bias was the main reason for influencing the validity of results of the Meta analysis, because it was the main basis for making a conclusion to obtain the published studies. Moreover, positive results were over-emphasized by some editors or in some magazines, so that some negative results were concealed. Meanwhile, a loss of literature could be caused by an insufficient retrieval method and various other reasons. In our study, the analysis of the effective rate showed no publication bias; however, since four other indices were limited by too small a sample size, the corresponding analysis was not made.

This systematic evaluation and Meta analysis were performed strictly according to the PRISMA Statement: it first evaluated the clinical efficacy and safety of Aspirin with Combined Compound Danshen Dropping Pills on the treatment in Middle-aged and Elderly Patients with CHD. it evaluated how standard the clinical trial was from a methodological point of view, and also offered a direction for further study. However, it is important to note that our study still had certain limitations: (1) The included literature was of low quality; the generation of random allocation sequences and the concealment of randomization protocols were not reported in some studies; the conditions of dropout and withdrawal from the study were not described in detail; certain problems occurred (such as missing study data), which influenced the evidence level and popularization degree. (2) Cases were not fully included according to generally recognized international diagnostic criteria; standard uniform outcome measurement indices were not observed; a subjective composite outcome scoring index was adopted and the evaluation criteria for efficacy was formulated by ourselves. (3) Treatment courses were shorter; there were no important clinical indices for long-time observation, and the superiority of TCM combine drugs in terms of overall efficacy could not be fully displayed. (4) The study have registered Prospero, but the registration is not approved yet. It is a concern as it introduces potential bias to the review and does not align with Cochrane guidance.

## Conclusion

At present, Aspirin with Combined Compound Danshen Dropping Pills in the treatment of CHD is a safer, more effective method of therapy for CHD. It has various advantages, such as high clinical effectiveness, TC, TG, and LDL-C decreased more significantly in the patients treated with DSP combined with aspirin, than in those treated by aspirin alone. It is worthy of clinical popularization and application. Since the original studies were of too low quality, a strict standard clinical trial should be made for verification in the future under the premise that the TCM characteristics is ensured.

## Data Availability Statement

The original contributions presented in the study are included in the article/Supplementary Material, further inquiries can be directed to the corresponding author/s.

## Author Contributions

ZL and GL took part in the design of the study, performed the literature survey, and drafted the manuscript. YWM took part data management implementation of the study. LL was responsible for statistical analysis and the methodological design of study. All authors have made substantive contributions to this study in regard to design and implementation. All the authors read and approved the final manuscript.

## Conflict of Interest

The authors declare that the research was conducted in the absence of any commercial or financial relationships that could be construed as a potential conflict of interest.
