# Supplementary material for: The Effects of Aspirin With Combined Compound Danshen Dropping Pills on Hemorheology and Blood Lipids in Middle-Aged and Elderly Patients With CHD: A Systematic Review and Meta-Analysis
Source: Front Public Health. 2021 Jun 18;9:664841. doi: 10.3389/fpubh.2021.664841 (PMC8249928; doi:10.3389/fpubh.2021.664841)
Supplement: Supplementary file 1 [file Data_Sheet_1.pdf]

Appendix：

Search strategies:

The search model was as follows:

1.Pubmed 2021/3/20

#1 search:((((Compound Danshen Dropping Pills[Title/Abstract]) OR (DSP[Title/Abstract])) OR (Danshen Dropping Pills [Title/Abstract])) OR（fufang danshen[Title/Abstract]))))

#2 search: （ salvia miltiorrhiza[Title /Abstract])OR (compound red-rooted salvia[Title/Abstract]）OR (acetylsalicylic acid[Title/Abstract]）

#3 search:((Coronary Heart Disease[Title/Abstract]) OR (CHD[Title/Abstract]))

#4 search: Blood Lipids[Title/Abstract]

#5 search: Hemorheology[Title/Abstract]

#6 search:Middle-aged[Title/Abstract]

#7search: Elderly[Title/Abstract]

#8 serach:"Blood Lipids" [MeSH Terms]

#9 search:"Hemorheology"[MeSH Terms]

#10 search:"Coronary Heart Disease"[MeSH Terms]

#11 search:"salvia miltiorrhiza"[MeSH Terms]

#12 search:"compound red-rooted salvia"[MeSH Terms]

#13 search:"acetylsalicylic acid"[MeSH Terms]

#14 search:"Compound Danshen Dropping Pills"[MeSH Terms]

#15 search: #1 OR #2 OR #3 OR #4 OR #5 OR #6 OR #7 OR #8 OR #9 OR #10 OR #11 OR #12 OR #13 OR #14

#16 search: ("2010/01/01"[Date-Publication]:"2020/03/20[Date-Publication])

2. CNKI 2021/3/20

#1 search:(((([SU=Compound Danshen Dropping Pills) OR (SU=DSP)) OR (SU=Danshen Dropping Pills)) OR (SU=fufang danshen))

#2 search:(((SU=Middle-aged)) OR (SU=Elderly))

#3 search: (((([SU=salvia miltiorrhiza) OR (SU=compound red-rooted salvia)) OR (SU=acetylsalicylic acid))

#4 search: (([SU=Coronary Heart Disease) OR (SU=CHD))

#5 search:SU=Blood Lipids

#6 search: SU=Hemorheology

#7 search: #1 OR #2 OR #3 OR #4 OR #5 OR #6

#8 search: ("2010/01/01"[Date-Publication]:"2020/03/20[Date-Publication])

1. TC group sensitivity analysis

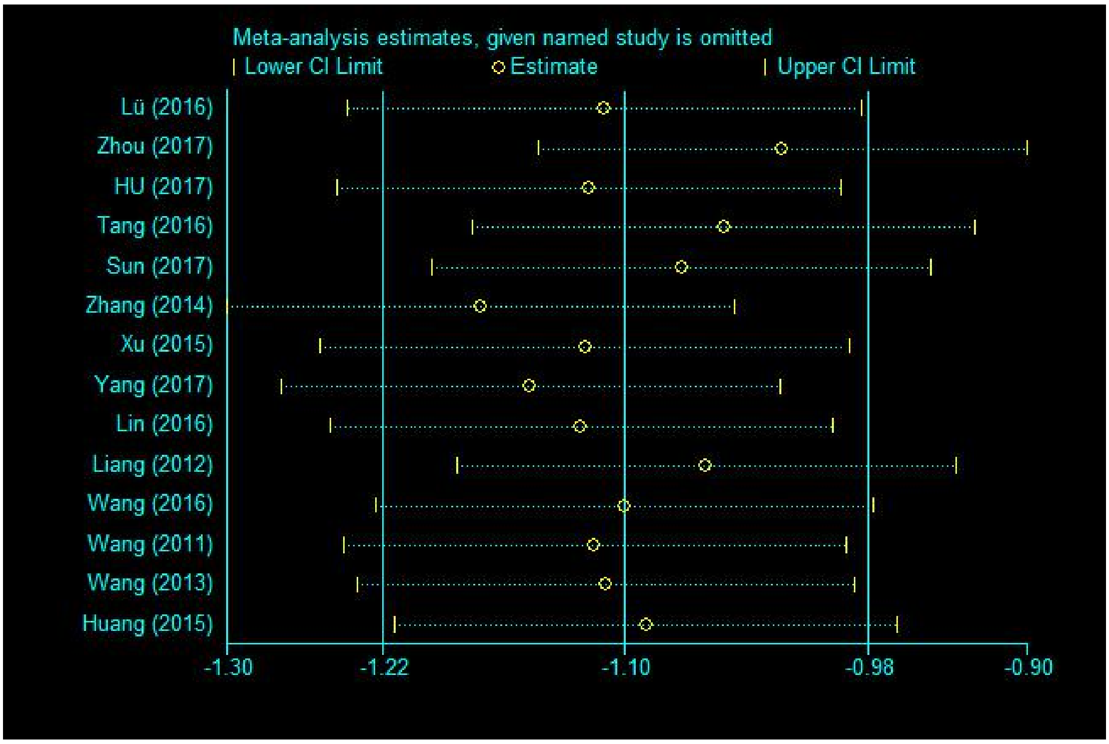

2. TC group sub-group analysis

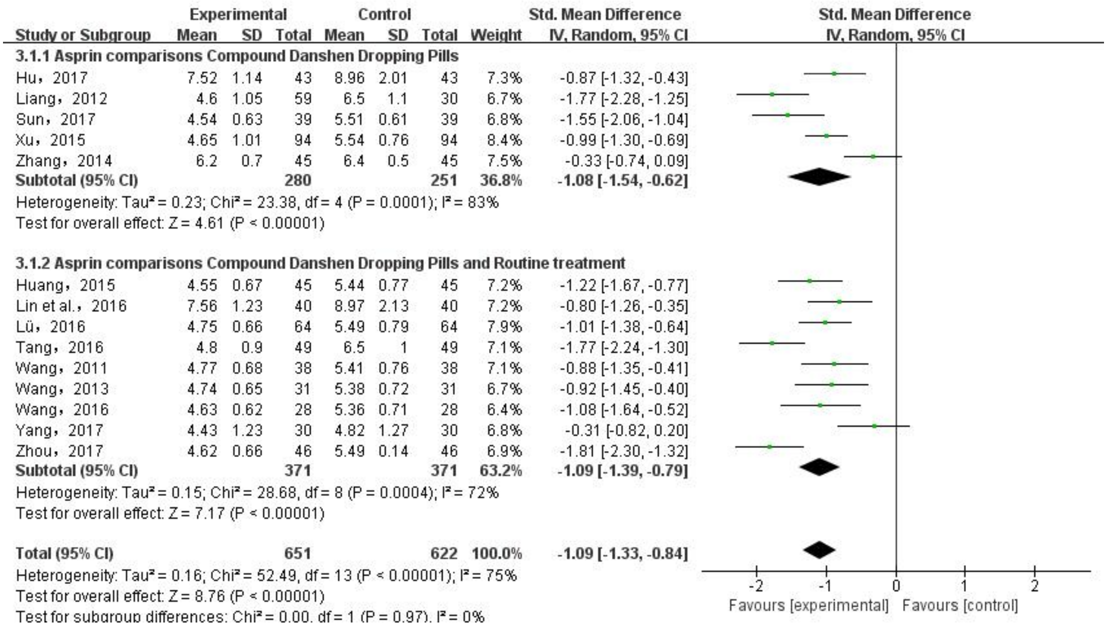

3. TG group sensitivity analysis

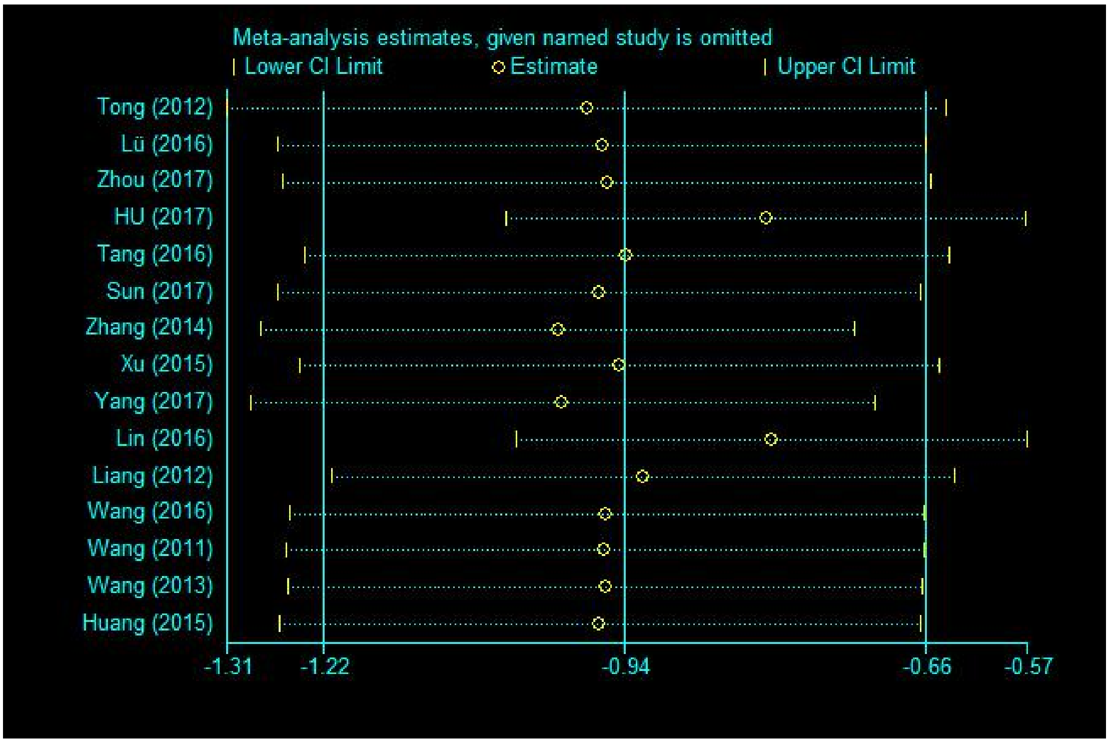

4. TG group sub-group analysis

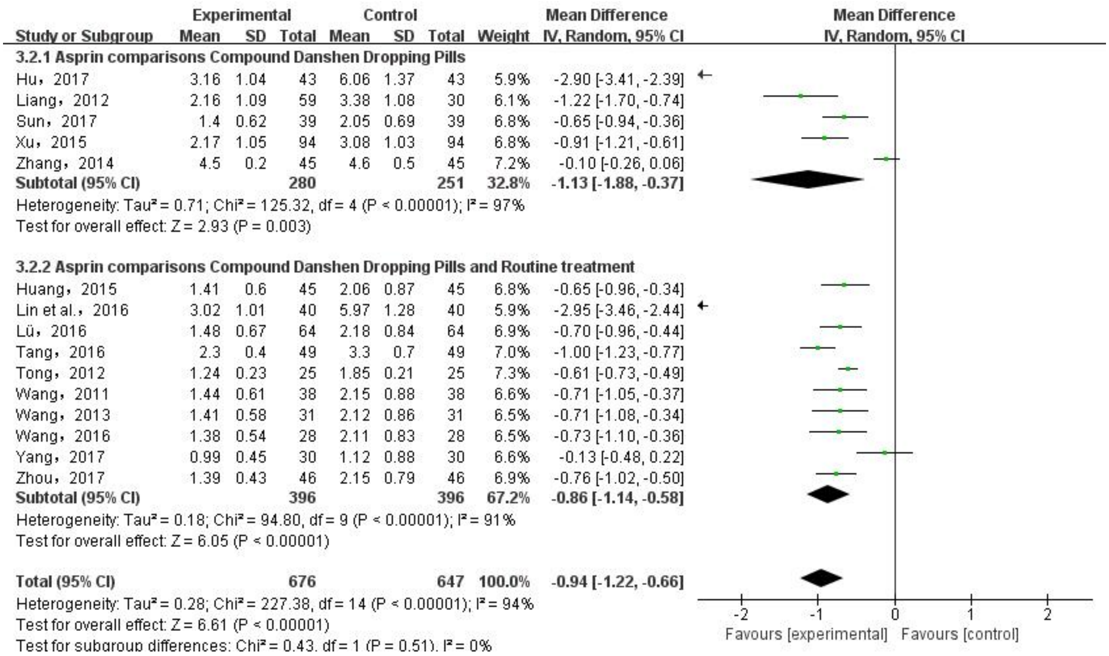

5. HDL-C group sensitivity analsysis

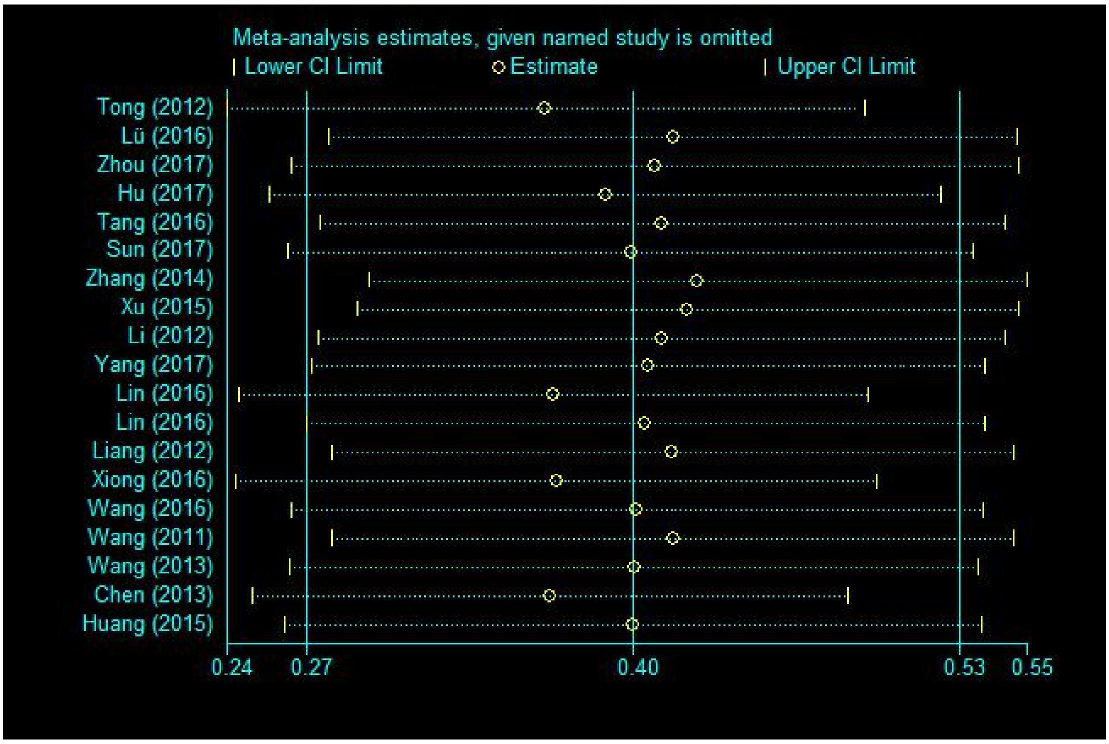

6. HDL-C group sub-group analysis

Study or Subgroup	Experimental Mean	Experimental SD	Experimental Total	Control Mean	Control SD	Control Total	Weight	Mean Difference IV, Random, 95% CI
**3.3.1 Asprin comparisons Compound Danshen Dropping Pills**								
Hu，2017	2.63	0.42	43	2.04	0.34	43	5.3%	0.59 [0.43, 0.75]
Liang，2012	1.58	0.33	59	1.45	0.34	30	5.4%	0.13 [-0.02, 0.28]
Lin，2016	2.2	0.56	39	1.24	0.33	39	5.1%	0.96 [0.76, 1.16]
Li，2012	1.25	0.42	50	1.04	0.34	50	5.4%	0.21 [0.06, 0.36]
Sun，2017	1.44	0.31	39	1.02	0.39	39	5.4%	0.42 [0.26, 0.58]
Xiong，2016	2.21	0.61	29	1.22	0.35	29	4.7%	0.99 [0.73, 1.25]
Xu，2015	1.5	0.34	94	1.47	0.31	94	5.7%	0.03 [-0.06, 0.12]
Zhang，2014	0.5	0.1	45	0.56	0.5	45	5.4%	-0.06 [-0.21, 0.09]
**Subtotal (95% CI)**			**398**			**369**	**42.5%**	**0.40 [0.16, 0.64]**
Heterogeneity: Tau² = 0.11; Chi² = 141.10, df = 7 (P < 0.00001); I² = 95%								
Test for overall effect: Z = 3.23 (P = 0.001)								
**3.3.2 Asprin comparisons Compound Danshen Dropping Pills and Routine treatment**								
Chen，2013	2.08	0.47	60	1.17	0.32	60	5.4%	0.91 [0.77, 1.05]
Huang，2015	1.45	0.3	45	1.04	0.27	45	5.6%	0.41 [0.29, 0.53]
Lin et al.，2016	2.37	0.52	40	2.06	0.51	40	4.9%	0.31 [0.08, 0.54]
Lü，2016	1.17	0.39	64	1.03	0.26	64	5.6%	0.14 [0.03, 0.25]
Tang，2016	1.6	0.4	49	1.4	0.4	49	5.4%	0.20 [0.04, 0.36]
Tong，2012	3.19	0.58	25	2.08	0.48	25	4.5%	1.11 [0.81, 1.41]
Wang，2011	1.16	0.35	38	1.03	0.29	38	5.4%	0.13 [-0.01, 0.27]
Wang，2013	1.41	0.32	31	1.01	0.27	31	5.4%	0.40 [0.25, 0.55]
Wang，2016	1.41	0.28	28	1.03	0.26	28	5.5%	0.38 [0.24, 0.52]
Yang，2017	1.82	0.64	30	1.56	0.71	30	4.1%	0.26 [-0.08, 0.60]
Zhou，2017	1.48	0.23	46	1.2	0.17	46	5.7%	0.28 [0.20, 0.36]
**Subtotal (95% CI)**			**456**			**456**	**57.5%**	**0.40 [0.25, 0.55]**
Heterogeneity: Tau² = 0.06; Chi² = 113.62, df = 10 (P < 0.00001); I² = 91%								
Test for overall effect: Z = 5.27 (P < 0.00001)								
**Total (95% CI)**			**854**			**825**	**100.0%**	**0.40 [0.27, 0.53]**
Heterogeneity: Tau² = 0.07; Chi² = 262.44, df = 18 (P < 0.00001); I² = 93%								
Test for overall effect: Z = 6.08 (P < 0.00001)								
Test for subgroup differences: Chi² = 0.00, df = 1 (P = 0.98), I² = 0%								

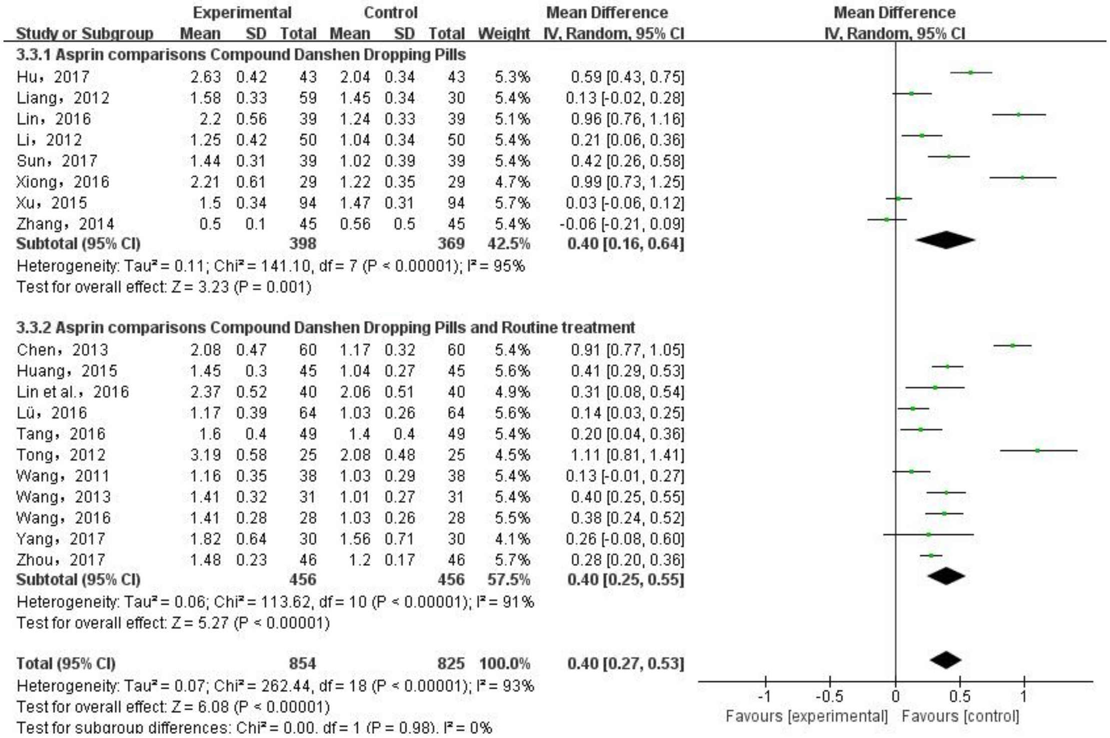

7. LDL-C group sensitivity analysis

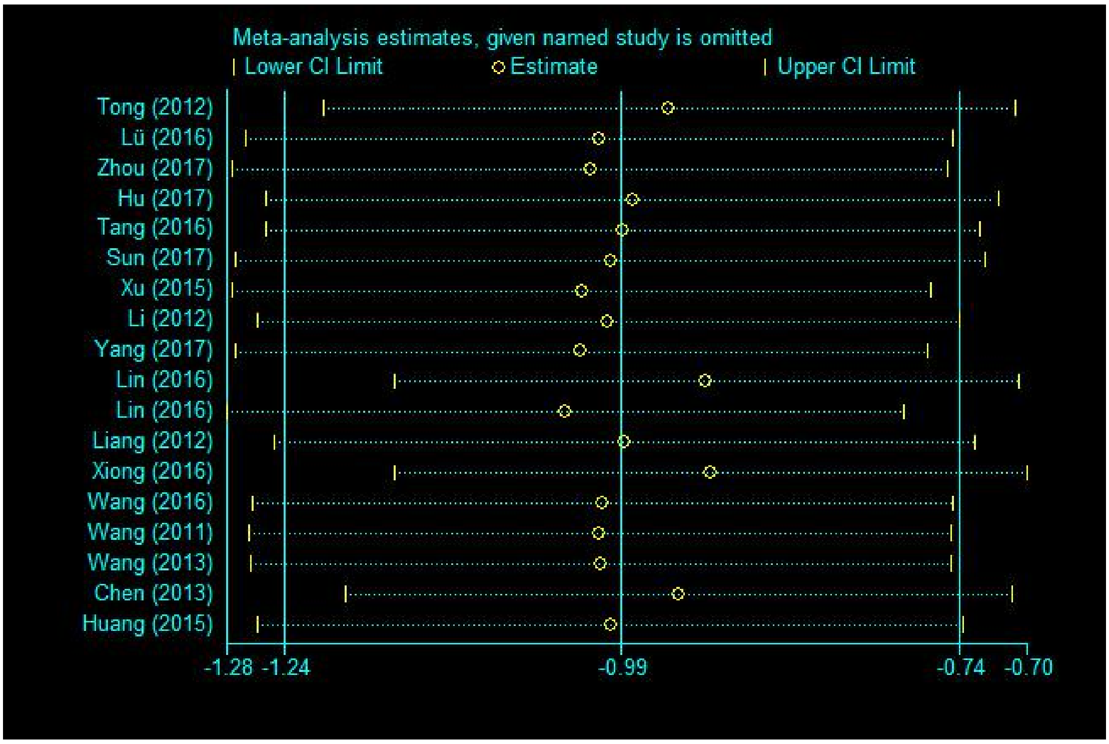

8. LDL-C group sub-group analysis

Study or Subgroup	Experimental Mean	Experimental SD	Experimental Total	Control Mean	Control SD	Control Total	Weight	Mean Difference IV, Random, 95% CI
**3.4.1 Asprin comparisons Compound Danshen Dropping Pills**								
Hu，2017	1.26	0.43	43	2.36	0.51	43	6.0%	-1.10 [-1.30, -0.90]
Liang，2012	3.59	1.08	59	4.62	1.1	30	5.0%	-1.03 [-1.51, -0.55]
Lin，2016	2.14	0.49	39	4.17	0.69	39	5.8%	-2.03 [-2.30, -1.76]
Li，2012	3.06	1.14	50	3.87	1.08	50	5.2%	-0.81 [-1.25, -0.37]
Sun，2017	3.11	0.34	39	3.95	0.37	39	6.0%	-0.84 [-1.00, -0.68]
Xiong，2016	2.12	0.43	29	4.22	0.71	29	5.7%	-2.10 [-2.40, -1.80]
Xu，2015	3.61	0.98	94	4.12	0.95	94	5.7%	-0.51 [-0.79, -0.23]
**Subtotal (95% CI)**			**353**			**324**	**39.4%**	**-1.21 [-1.65, -0.76]**
Heterogeneity: Tau² = 0.33; Chi² = 118.41, df = 6 (P < 0.00001); I² = 95%								
Test for overall effect: Z = 5.35 (P < 0.00001)								
**3.4.2 Asprin comparisons Compound Danshen Dropping Pills and Routine treatment**								
Chen，2013	2.52	0.49	60	4.18	0.68	60	5.9%	-1.66 [-1.87, -1.45]
Huang，2015	3.12	0.85	45	3.96	0.91	45	5.5%	-0.84 [-1.20, -0.48]
Lin et al.，2016	1.8	0.57	40	2.15	0.46	40	5.9%	-0.35 [-0.58, -0.12]
Lü，2016	3.12	0.85	64	3.82	0.97	64	5.6%	-0.70 [-1.02, -0.38]
Tang，2016	3.6	0.7	49	4.6	0.8	49	5.7%	-1.00 [-1.30, -0.70]
Tong，2012	2.35	0.48	25	3.92	0.61	25	5.7%	-1.57 [-1.87, -1.27]
Wang，2011	3.15	0.91	38	3.85	0.93	38	5.3%	-0.70 [-1.11, -0.29]
Wang，2013	3.12	0.87	31	3.82	0.91	31	5.2%	-0.70 [-1.14, -0.26]
Wang，2016	3.06	0.85	28	3.79	0.89	28	5.1%	-0.73 [-1.19, -0.27]
Yang，2017	1.88	0.76	30	2.28	1.29	30	4.8%	-0.40 [-0.94, 0.14]
Zhou，2017	3.1	0.56	46	3.72	0.05	46	6.0%	-0.62 [-0.78, -0.46]
**Subtotal (95% CI)**			**456**			**456**	**60.6%**	**-0.85 [-1.15, -0.56]**
Heterogeneity: Tau² = 0.22; Chi² = 109.27, df = 10 (P < 0.00001); I² = 91%								
Test for overall effect: Z = 5.66 (P < 0.00001)								
**Total (95% CI)**			**809**			**780**	**100.0%**	**-0.99 [-1.24, -0.74]**
Heterogeneity: Tau² = 0.26; Chi² = 243.79, df = 17 (P < 0.00001); I² = 93%								
Test for overall effect: Z = 7.86 (P < 0.00001)								
Test for subgroup differences: Chi² = 1.69, df = 1 (P = 0.19), I² = 40.7%								

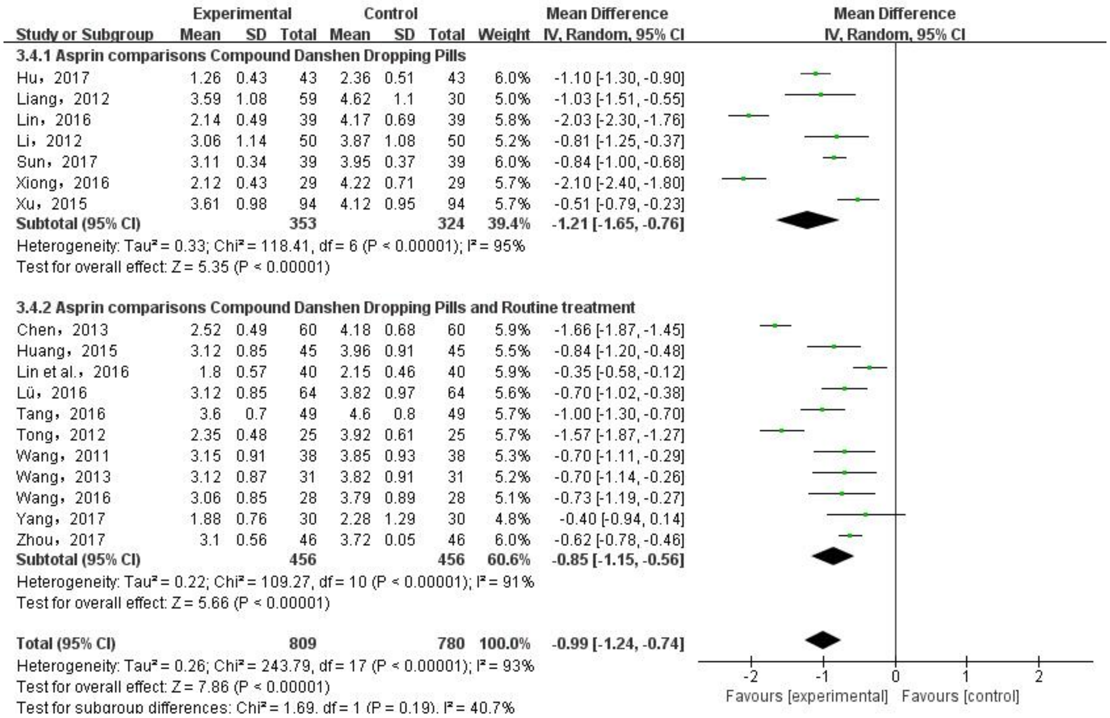

9. High shear viscosity group sub-group analysis

Study or Subgroup	Experimental Mean	Experimental SD	Experimental Total	Control Mean	Control SD	Control Total	Weight	Mean Difference IV, Random, 95% CI
**3.6.1 Asprin comparisons Compound Danshen Dropping Pills**								
Hong，2015	5.3	0.5	64	5.4	0.7	64	25.2%	-0.10 [-0.31, 0.11]
Liang，2012	3.45	0.28	59	5.95	0.89	30	24.9%	-2.50 [-2.83, -2.17]
Xu，2015	3.78	0.53	94	5.35	0.91	94	25.2%	-1.57 [-1.78, -1.36]
**Subtotal (95% CI)**			**217**			**188**	**75.3%**	**-1.39 [-2.70, -0.07]**
Heterogeneity: Tau² = 1.33; Chi² = 175.82, df = 2 (P < 0.00001); I² = 99%								
Test for overall effect: Z = 2.06 (P = 0.04)								
**3.6.2 Asprin comparisons Compound Danshen Dropping Pills and Routine treatment**								
Yang，2017	4.53	0.69	30	4.81	0.77	30	24.7%	-0.28 [-0.65, 0.09]
**Subtotal (95% CI)**			**30**			**30**	**24.7%**	**-0.28 [-0.65, 0.09]**
Heterogeneity: Not applicable								
Test for overall effect: Z = 1.48 (P = 0.14)								
**Total (95% CI)**			**247**			**218**	**100.0%**	**-1.11 [-2.18, -0.05]**
Heterogeneity: Tau² = 1.16; Chi² = 193.19, df = 3 (P < 0.00001); I² = 98%								
Test for overall effect: Z = 2.05 (P = 0.04)								
Test for subgroup differences: Chi² = 2.52, df = 1 (P = 0.11), I² = 60.2%								

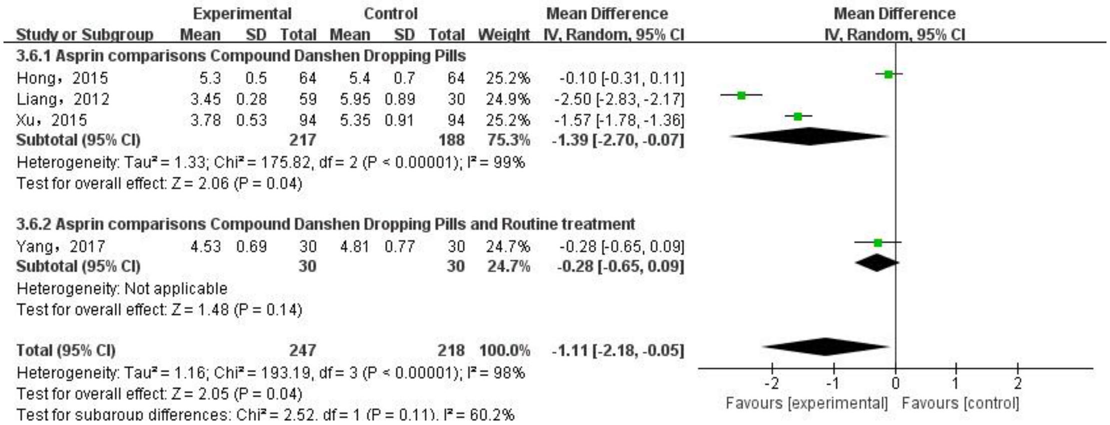

10. PAMG group sub-group analysis

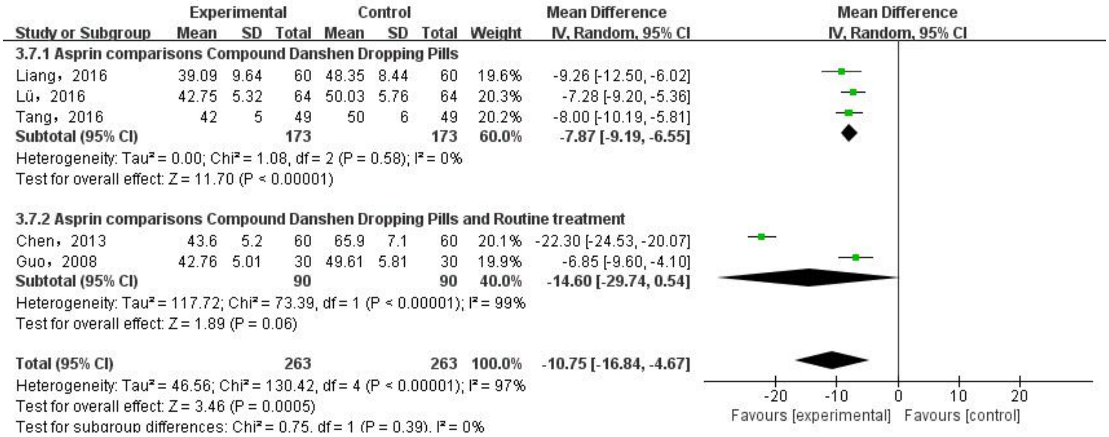

Patient and Public Involvement:

No patient involved.

Data Availability Statement

No additional data available.

Author contributions:

All authors have made substantive contributions to this study in regard to design and implementation. Z.L.,G.L. took part in the design of the study,performed the literature survey and drafted the manuscript. YW.M. took part Data Management implementation of the study. Z.L. was responsible for statistical analysis. Z.L. was responsible for the methodological design of study.L.L.&. Z.L. is the corresponding authors for the article. All the authors read and approved

the final manuscript.

Competing interests:

Te authors declare that they have no competing interests, and due care has been taken to ensure the integrity of the work. Neither the entire paper nor any part of its content has been published or has been accepted elsewhere. It is not being submitted to any other journal. The authors have no other relevant affiliations or financial involvement with any organization or entity with a financial interest in or financial conflict with the subject matter or materials discussed in the manuscript apart from those disclosed.

Funding

We gratefully acknowledged the cooperation of all research staff and participants.Te funding was supported by the National Natural Science Foundation of China (No.81503456), and the Tianjin enterprise science and technology commissioner project (No.18JCTPJC65500).

Compliance with ethics guidelines

This manuscript is a review article and does not involve a research protocol requiring approval by the corresponding institutional review board or ethics committee.
